# Malpractice

**Published:** 1894-12

**Authors:** 


ARTICLE VI.

### MALPRACTICE.

With profit the motto of the great lawyer, David Paul Brown, could be adopted by the medical and surgical profession: A doctor who knows nothing of law, and a lawyer who knows nothing of medicine, are deficient in essential requisites of their respective professions."

Medical men and surgeons are vitally interested in whatever relates to their liabilities as practitioners. The busy practitioner—and he is generally busy in proportion to his zeal, ability and competency for his work—has little time to devote to the long-drawn-out and very generally confused treatise on medico-legal questions contained in books. The gist of what he wants, what he needs, is, what are his duties and liabilities, where do they begin and where end, what constitutes legal rights, wrongs and remedies as they relate to his profession. It has been too frequent, and yet is, that criminal or civil responsibility for omission or commission has been charged to innocent practitioners.

In a not very distant past so great were the facilities, so strong the encouragement of lawyers who shared in the spoils, to brlng suits for malpractice, that able surgeons would not risk this constant menace by the practice of surgery. They would practice medicine but not surgery, for the reason of the dangers attending its practice. They turned in alarm from the risks of having the accumulations of years of labor wrested from them by ruinous litigation. These suits were more frequent in the past than now. A just and enlightened judiciary and a united profession has largely diminished the number of these cases, instituted, as they about always are, from avarice, malice or ignorance.

There are, however, many prosecutions instituted that never find their way into legal reports; they are only locally heard of through newspapers of narrow neighborhood circulation, and only serve to startle practitioners of a limited section with the perils of medical and surgical practice. The charges of learned judges and the decisions of the higher courts have laid down the underlying principles of these cases with such clearness and emphasis as to do more than merely discourage their institution, even to condemn them in many instances as vexations and cruelly unjust. The number of these cases will continne to decrease with the advance of medicolegal knowledge in the professions of both law and medicine, and with the maintenance of a high standard of anatomical, physiological and pathological knowledge in the latter profession.

Of the many embarrassments which the physician has to confront in general practice, tbere is none greater or that so severely tests professional merit and character, that so endangers reputation and to which he is so averse, as being called into the courts, whether of criminal or civil law.

There is one fact of easy recognition. That is, that more is expected of physicians—that they are more subjected to indiscriminate, ill-considered and unsparing criticism than any other class of men. When errors or mistakes are made they are noised abroad, passed from neighbor to neighbor, from one acquaintance to another, and not infrequently heralded through the newspapers. The attempt is often to hold them legally liable for what no human skill could avoid or human foresight anticipate. Nothing is allowed for the complex character of the trouble with which they have to deal, the hidden influence which baffles the best science.

Latent conditions often give the most skilful of surgeons bad results. For these he is sometimes carried into vexations and expensive litigation through the counsel of some unscrupulous lawyer ignorant of the principles governing malpractice cases.

Medicine and surgery have emerged from many forms of barbarism and superstition, and so have the law courts from barbarous rulings and decisions in malpractice cases. The real, inherent difficulties of the profession have grown to be better understood by the law courts. It is recognized that there is very much to qualify results in any given case. An eminent authority condenses much truth in the following: "A source of uncertainty in the practical art of medicine is the difficulty we find in applying to new cases the knowledge we have acquired from observation. This application is made upon the principle of either experiment or analogy.

"We are said to proceed upon experience when the circumstances in the new case are the same as in those caes from which our knowledge was derived. When the circumstances are not the same, but similar, we proceed upon analogy, and our confidence in the result is weaker than when we proceed on experience. The more numerous the points of resemblance are, the greater is our confidence, because it approaches the more nearly to that which we derive from experience; and the fewer the points of resemblance, the confidence we feel is more and more diminished. When in the practice of medicine we apply to new cases the knowledge acquired from others, which we believe to have been of the same nature, the difficulties are so great that it is doubtful whether in any case we can properly be said to act upon experience, as we do in other departments of science; for we have not the means of determining with certainty that the condition of the disease, the habits of the patient, and all the circumstances that enter into the character of the affection are precisely the same in the two cases; and if they differ in any one particular, we cannot be said to act from experience but from analogy. The difficulties and sources of uncertainties that meet us at every stage of such investigations are, in fact, so great and numerous that those who have had the most extensive opportunities of observation will be the first to acknowledge that our pretended experience must, in general, sink into analogy, and even our analogy too often into conjecture.

As these difficulties and embarrassments grow to be better understood by courts and by jurymen who come from the body of our committes, the fewer will be malpractice suits, and the more frequent the verdict "not guilty."

The rigid enforcement of the old rule that the practitioner must apply, without mistake, what is settled in the profession, was, up to a very recent date, a fruitful source of suits for malpractice. This rule has been greatly modified under the light of the best modern judicial authorities. Progress in modern medicine and surgery has pushed aside many old practices as useless and in many instances injurious. Especially is there allowed greater room for difference of opinion in the exercise of the art of surgery, for there are usually several ways for doing the same thing, different operations for the same injuries, each operator having a partiality for his own methods, those by which he has secured the best results.

One of the best legal authorities on the law of malpractice very aptly says: "It is not enough for a surgeon to plead that his treatment was that taught him by the ablest members of the profession and the best schools twenty-five years ago, because in a science that is advancing with the rapidity of medicine and surgery—that is, by observation and experience, yearly, and almost daily, correcting errors in practice and abandoning hoary-headed theories, the fallacy of which has become apparent, upon which the practice has heretofore been based—that is receiving auxiliary agencies from all the rapidly advancing sister sciences—there will be new facilities afforded in practice year by year and errors constantly exploded. The authority, therefore, that was at a previous day considered good, and upon which the courts acted, may not at this time be admitted as the present standard of knowledge required of the physician and surgeon. Old physicians and surgeons cannot, therefore, rely with safety upon their elementary education and what they may have learned in practice. It is absolutely important for the protection of the patient as well as of the surgeon, if he assumes the responsibility of performing an operation fraught with so great interest, that he should make use of every reasonable means of knowing what is considered the best treatment at the time of the operation—not what would have been the proper course twenty years ago. A medical man cannot, with any safety or propriety, practice year after year without keeping himself informed as to the improvements of his science, especially if he practice surgery."

The responsibilities and liabilities of the profession are not in any strict sense diminishing; they broaden with the light and advance of the profession. The law keeps pace in its requirements.

Responsibility is measured by the professed ability to accomplish. Where a large price is demanded and paid for services, there is a corresponding increase of responsibility, there being an implied claim of superiority of knowledge and skill over others. And this responsibility is only reduced or relieved by the operation of cause or influences beyond the physician's or surgeon's detection, or over which he has no control.

Penal laws are made to protect society against the charlatan and the ignorance of the quack. For an educated, reputable and honorable physician to compromise such cases is an implied consent to be classed with those professional pests; is to compromise personal honor and the honor of the profession. Our judicial tribunals will do no more than that which every educated and honorable physician and surgeon will demand, that the severest judgment of the law should be inflicted upon those who are ignorant or recklessly trifle with health and human life.

Every detail of professional conduct, upon which is founded the good name and repute of the physician and surgeon, receives the severest of judicial scrutiny in all malpractice suits. And there is accountability in damages, measurable with the extent of the evil results, for ignorant, careless or unprofessional conduct. One of the ablest of English surgeons says: "Genius for our art may shine out on great occasions, and brilliant devices contend against remarkable deviations from health, but *conduct* is *required* of us all. The word *conduct* has a wide interpretation; it appeals to the application of the humanities of life, as well as the exercise of skill and industry in the application of our best resources in the treatment of disease."

The law regards the undertaking to be only for the use of proper means. It is not regarded that the cure is in the physician, but is largely dependent upon the constitution of the patient and the influences of causes beyond his control. In one of the oldest reported English cases [1767) the Lord Chief Justice said: "Ignorance is rashness. A physician may intend well and yet his conduct be so exceptional that he must be held liable, criminally when life is lost by such conduct, though he did not intend it at the time."

Though extraordinary skill is not required when the act to be done depends on the skill of the operator alone, the law will imply an engagement to use that skill and to produce the desired result, from the employment of one professing it and holding himself out to the world as having it. The errors for which he may be held liable may be either of omission or commission. He is not responsible for those disasters which are not the result of his own conduct, or for those the result of the refractory conduct of the patient, upon whom rests the collateral duties of submitting to the requirements of his professional attendant.

He is liable for any unnecessary pain or delay occasioned by the application of unskillful and improper remedies. Amputations, fractures and dislocation, and deformities arising therefrom, are made the grounds of many malpractice suits.

In the cases of amputation a legal text writer puts the subject in strong form: Under the state of things one of the most difficult and close questions of surgery arises—a question upon the solution of which depends the life of the patient, perhaps, and possibly the reputation of the surgeon— to decide which question correctly calls into requisition correct logic, close observation and extensive surgical knowledge; and that is the question of *amputation*. In these difficult cases the result, let it be for or against amputation, will generally be critically questioned. The loss of a limb, on the one hand, will raise the question of the propriety of the amputation long after the real condition that was supposed to demand it is forgotten by all, perhaps, except the surgeon himself. If amputation is overruled and the patient dies, the surgeon is blamed for the sad result. These cases are fruitful sources of litigation. If the limb was amputated, it is said there was gross carelessness or recklessness and a want of care and skill—that the limb might have been saved had proper effort been made at the right time. If the surgeon gives to the patient the benefit of a doubt as to the propriety of the amputation in a close case, and after long and vigilant watching surmounts great difficulties saves the limb in a condition to be of great use to the patient, yet, it not being a perfect cure, then the surgeon is said still to be at fault, notwithstanding the injury was so severe that the question of amputation hung evenly in the balance for a time, and he is sued.

Such cases are familiar to the profession.—Editorial in *Med. and Surg. Reporter*.

